# Sex-related differences in the efficacy of immune checkpoint inhibitors in malignancy: a systematic review and meta-analysis

**DOI:** 10.18632/aging.203100

**Published:** 2021-06-04

**Authors:** Li-Ting Lai, Wei-Guo Gu, Ming-Bin Hu, Wei-Jia Wang, Shan-Shan Wang, Ya-Jun Huai, Jin-Hong Mei, Chun-Liang Wang

**Affiliations:** 1 Department of Oncology, The First Affiliated Hospital of Nanchang University, Nanchang, Jiangxi, China; 2 Department of Pathology, The First Affiliated Hospital of Nanchang University, Nanchang, Jiangxi, China; 3 Department of Neurosurgery, The First Affiliated Hospital of Nanchang University, Nanchang, Jiangxi, China

**Keywords:** immunotherapy, cancer, therapeutic efficacy, sex-specific differences, meta-analysis

## Abstract

Although disease susceptibility is known to differ between men and women, it is controversial whether the efficacy of immune checkpoint inhibitors for malignancies also differs between the sexes. We conducted a meta-analysis to explore the impact of sex on immune checkpoint inhibitor treatment outcomes. We searched PubMed, Embase and the Cochrane Library databases from inception to October 1, 2020 for randomized controlled trials of immune checkpoint inhibitors with hazard ratios (HRs) stratified by sex. We calculated the pooled HRs for men and women using the ln(HR), and assessed the heterogeneity between the two estimates through an interaction test. In total, 22,268 patients from 39 randomized controlled trials were included. Immune checkpoint inhibitors yielded better overall survival than conventional agents in both men (HR: 0.75, 95% confidence interval [CI]: 0.71–0.80) and women (HR: 0.77, 95% CI: 0.70–0.85). Progression-free survival benefits were also observed in both men (HR: 0.64, 95% CI: 0.58–0.70) and women (HR: 0.67, 95% CI: 0.58–0.77) treated with immune checkpoint inhibitors. No sex differences in the response to immune checkpoint inhibitors were found when overall survival and progression-free survival were used as the endpoints.

## INTRODUCTION

Polymorphisms and other variations in genes encoding immune proteins may cause sex differences in immunity [1] and immune-related disease susceptibility [2, 3], whether the genes are expressed on sex chromosomes or autosomes. In insects, birds, mammals and other species, males tend to have lower innate and adaptive immune responses than females, and thus differ in their exposure to, recognition of, removal of and spread of pathogenic microorganisms. In general, women produce higher levels of basal immunoglobulin and antibodies against pathogens and vaccines than men. Further, CD3+ and CD4+ T cell counts, CD4+ to CD8+ T cell ratios and Th1 responses are higher in women than in men [1, 4, 5].

Men generally are at higher risk for malignancies than women. Moreover, the cancer-related mortality rate is almost two times higher in men than in women, with sex differences being most significant in laryngeal, esophageal, bladder and lung cancers [6, 7]. On the other hand, women account for 80% of all autoimmune disease cases worldwide, including Sjogren’s syndrome, systemic lupus erythematosus, scleroderma, thyroid disease and myasthenia gravis [8]. These sex differences reflect the effects of hormones, genes and the environment on the immune system, which can change throughout a person’s life [1, 9].

Immunosuppression and the escape of malignant tumor cells are key events in carcinogenesis [10]. These processes are controlled by immune checkpoints (a series of co-stimulatory and co-inhibitory receptors and their ligands), among which the cytotoxic T-lymphocyte-associated protein 4 (CTLA-4) and programmed cell death 1 (PD-1)/programmed cell death 1 ligand 1 (PD-L1) pathways are significant therapeutic targets. These pathways are important for immune homeostasis under physiological conditions, but also can be mechanisms whereby carcinoma cells escape immune surveillance. Monoclonal antibodies have been developed against PD-1/PD-L1 (e.g., pembrolizumab, nivolumab, tezolizumab, aurumab and durvalumab) and CTLA-4 (e.g., ipilimumab and tremelimumab), and their clinical application has launched a new era of cancer treatment [11, 12].

Sex hormones regulate the PD-1/PD-L1 signaling pathway, and may influence immune function by enhancing the PD-1 co-stimulatory pathway [13]. In animal models, sex differences have been observed in tumor immunity and immunotherapy responses [14]. However, an *in vivo* study on cytotoxic T cell-suppressing agents revealed no differences in their inhibition of either immunity or lymphocyte proliferation between men and women [15]. In a study evaluating prednisolone kinetics and responses, women exhibited a lower clearance, higher systemic exposure and higher distribution volume than men after a single oral dose; nevertheless, these pharmacokinetic changes did not lead to sex differences in the overall response to prednisolone [16].

Few studies have evaluated pharmacodynamics based on sex, especially for oncology agents. A meta-analysis by Conforti et al. [17] indicated that male patients benefited more from immunotherapy than female patients; however, another analysis found that the effects of anti-PD-1 and anti-CTLA-4 treatments in advanced cancer patients did not differ significantly between the sexes [18]. Thus, it remains to be clarified whether there are a sex-related differences in the therapeutic benefits of cancer treatments.

In this systematic review and meta-analysis, we explored the impact of sex on immune checkpoint inhibitor treatment outcomes in cancer patients. We hypothesized that the efficacy of immune checkpoint inhibitors would not depend on sex.

## RESULTS

### Study selection

Our initial search strategy identified 14,213 articles. After screening the articles based on their titles and abstracts, we excluded 13,865 studies that did not conform to the inclusion criteria. Then, we carefully scanned through the full texts of the remaining 348 articles, and selected 39 studies [19–57] for the final analysis. Among these, 33 studies [19–46, 53–57] provided overall survival (OS) data for men and women, and were used in the qualitative analysis of OS. In addition, 20 of the studies [21, 22, 27, 35–39, 42, 45, 47–56] reported data on progression-free survival (PFS) by sex, and were used for the qualitative analysis of PFS. Supplementary Figure 1 displays the research selection flowchart.

### Characteristics of the included studies

The 39 randomized controlled trials included in this study involved a total of 22,268 advanced cancer patients, of whom 15,314 (69%) were male and 6,954 (31%) were female. The main characteristics of the included studies are shown in Table 1. All the studies were multicenter randomized controlled trials published between 2010 and 2020. The number of subjects per study ranged from 120 to 1,274. There were 34 phase III trials, four phase II trials and one phase II/III trial (Table 1).

**Table 1:** Characteristics of the included randomized controlled trials.

**Study**	**Phase**	**Tumour type**	**Treatment groups**	**Patients**	**Number of men (%)**	**Number of women (%)**
Hodi et al. (2010)	3	Melanoma	Ipilimumab plus gp100 vs gp100; ipilimumab vs gp100	676	401 (59%)	275 (41%)
Robert et al. (2011)	3	Melanoma	Ipilimumab plus dacarbazine vs dacarbazine plus placebo	502	301 (60%)	201 (40%)
Robert et al. (2015)(1)	3	Melanoma	Pembrolizumab every 2 weeks vs ipilimumab; pembrolizumab every 3 weeks vs ipilimumab	834	497 (60%)	337 (40%)
Hodi et al. (2016)	2	Melanoma	Ipilimumab plus nivolumab vs ipilimumab plus placebo	142	95 (67%)	47 (33%)
Robert et al. (2015)(2)	3	Melanoma	Nivolumab vs dacarbazine	418	246 (59%)	172 (41%)
Larkin et al. (2018)	3	Melanoma	Nivolumab vs chemotherapy	405	261 (64%)	144 (36%)
Ribas et al. (2013)	3	Melanoma	Tremelimumab vs chemotherapy	655	372 (57%)	283 (43%)
Reck et al. (2016)(1)	3	Small-cell lung cancer	Ipilimumab plus etoposide plus platinum vs placebo plus etoposide plus platinum	954	643 (67%)	311 (33%)
Carbone et al. (2017)	3	NSCLC	Nivolumab vs chemotherapy	541	332 (61%)	209 (39%)
Reck et al. (2016)(2)	3	NSCLC	Pembrolizumab vs chemotherapy	305	187 (61%)	118 (39%)
Herbst et al. (2016)	2/3	NSCLC	Pembrolizumab (2 mg/kg) vs pembrolizumab (10 mg/kg) vs docetaxel	1033	634 (61%)	399 (39%)
Brahmer et al. (2015)	3	NSCLC	Nivolumab vs docetaxel	272	208 (76%)	64 (24%)
Govindan et al. (2017)	3	NSCLC	Ipilimumab plus chemotherapy vs chemotherapy	749	635 (85%)	114 (15%)
Borghaei et al. (2015)	3	NSCLC	Nivolumab vs docetaxel	582	319 (55%)	263 (45%)
Maio et al. (2017)	2b	Mesothelioma	Tremelimumab vs placebo	571	434 (76%)	137 (24%)
Motzer et al. (2015)	3	Renal cell carcinoma	Nivolumab vs everolimus	821	619 (75%)	202 (25%)
Bellmunt et al. (2017)	3	Urothelial carcinoma	Pembrolizumab vs chemotherapy	542	402 (74%)	140 (26%)
Ferris et al. (2016)	3	Head and neck squamous-cell carcinoma	Nivolumab vs chemotherapy	361	300 (83%)	61 (17%)
Cohen et al. (2018)	3	Head and neck squamous-cell carcinoma	Pembrolizumab vs chemotherapy	495	412 (83%)	83 (17%)
Kang et al. (2017)	3	Gastric or gastro-oesophageal junction cancer	Nivolumab vs placebo	493	348 (71%)	145 (29%)
Paz-Ares et al. (2018)	3	NSCLC	Pembrolizumab puls chemotherapy vs chemotherapy	559	455 (81%)	104 (19%)
Gandhi et al. (2018)	3	NSCLC	Pembrolizumab puls chemotherapy vs chemotherapy	616	363 (59%)	253 (41%)
West et al. (2019)	3	NSCLC	Atezolizumab puls chemotherapy vs chemotherapy	679	400 (59%)	279 (41%)
Antonia et al. (2018)	3	NSCLC	Chemoradiotherapy plus durvalumab vs Chemoradiotherapy	713	500 (70%)	213 (30%)
Horn et al. (2018)	3	Small-cell lung cancer	Atezolizumab puls chemotherapy vs chemotherapy	403	261 (65%)	142 (35%)
Mok et al. (2019)	3	NSCLC	Pembrolizumab vs chemotherapy	1274	902 (71%)	372 (29%)
Rittmeyer et al. (2017)	3	NSCLC	Atezolizumab vs docetaxel	850	520 (61%)	330 (39%)
Barlesi et al. ((2018)	3	NSCLC	Avelumab vs chemotherapy	529	367 (69%)	162 (31%)
Jotte et al. (2020)	3	NSCLC	Atezolizumab puls chemotherapy vs chemotherapy	683	557 (82%)	126 (18%)
Kato et al. (2019)	3	Oesophageal squamous cell carcinoma	Nivolumab vs chemotherapy	419	364 (87%)	55 (13%)
Shitara et al. (2018)	3	Gastric or gastro-oesophageal junction cancer	Pembrolizumab vs paclitaxel	395	286 (72%)	109 (28%)
Finn et al. (2019)	3	Hepatocellular carcinoma	Pembrolizumab vs placebo	413	338 (82%)	75 (18%)
Huang et al. (2020)	3	Oesophageal squamous cell carcinoma	Camrelizumab vs chemotherapy	448	400 (89%)	48 (11%)
Papadimitrak-opoulou et al. (2018)	3	NSCLC	Atezolizumab puls chemotherapy vs chemotherapy	578	384 (66%)	194 (34%)
Ribas et al. (2015)	2	Melanoma	Pembrolizumab (2 mg/kg) vs pembrolizumab (10 mg/kg) vs chemotherapy	361	213 (59%)	148 (41%)
Hellmann et al. (2018)	3	NSCLC	Nivolumab vs ipilimumab	299	204 (68%)	95 (32%)
Motzer et al. (2019)	3	Renal cell carcinoma	Avelumab plus axitinib vs sunitinib	886	660 (74%)	226 (26%)
Ascierto et al. (2019)	2	Melanoma	Pembrolizumab plus dabrafenib plus trametinib vs placebo plus dabrafenib plus trametinib	120	69 (58%)	51 (42%)
Socinski et al. (2018)	3	NSCLC	Atezolizumab puls chemotherapy plus bevacizumab vs chemotherapy plus bevacizumab	692	425 (61%)	267 (39%)

Abbreviation: NSCLC: non–small-cell lung cancer.

Seventeen studies were carried out in non-small cell lung cancer (NSCLC) patients; nine in melanoma patients; two each in gastric or gastro-esophageal junction carcinoma, head and neck squamous cell cancer, small cell lung cancer, esophageal squamous cell carcinoma and renal cell carcinoma patients; and one each in mesothelioma, hepatocellular carcinoma and urothelial carcinoma patients. Among the 22,268 subjects, 4,113 (14%) were melanoma patients and 10,954 (36%) were NSCLC patients. All the studies were performed in metastatic settings. No trial using PD-1/PD-L1 or CTLA-4 monoclonal antibodies as an intervention in patients with early malignant tumors reported the HRs for mortality outcomes by sex. Participants in the intervention group received pembrolizumab in 12 studies, nivolumab in 10 studies, atezolizumab in 6 studies, tremelimumab in 2 studies, avelumab in 2 studies, camrelizumab in 1 study and durvalumab in 1 study.

The primary endpoint for all eligible studies was PFS or OS (reviewed by an independent center for blinding). Random sequences were generated in all trials. The included studies were found to have moderate to good methodological quality (Supplementary Figure 2, Supplementary Figure 3). The main problem affecting the quality of the research was lack of blinding, as some of the studies were open-labeled (no blinding of participants and personnel) rather than double-blinded [31, 37].

### OS analysis

**Figure 1:**
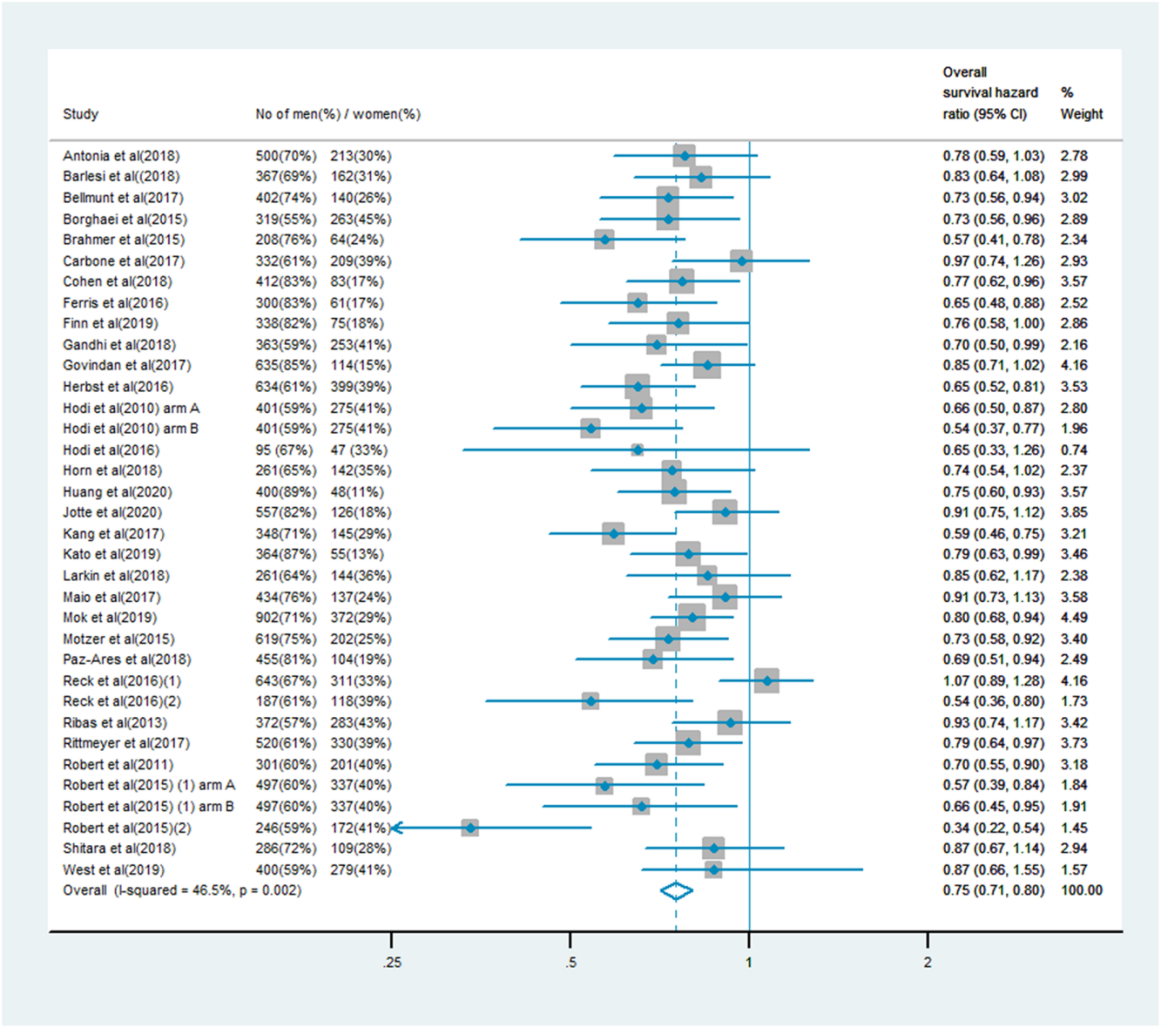
Overall survival hazard ratio in male patients in the immune checkpoint inhibitor group compared with the control group.

**Figure 2:**
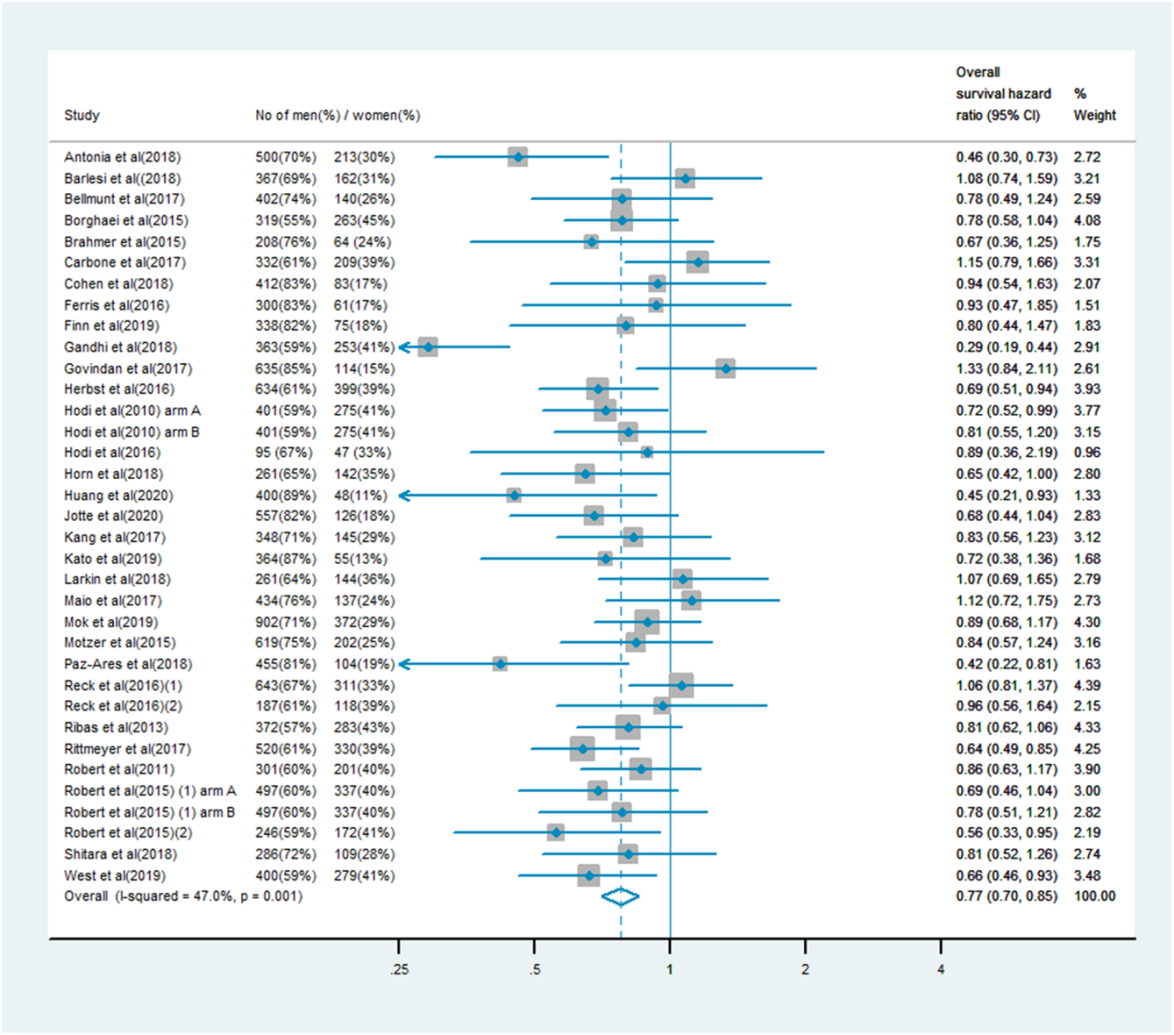
Overall survival hazard ratio in female patients in the immune checkpoint inhibitor group compared with the control group.

The analysis of OS included 19,332 patients, of whom 13,359 (69%) were male and 5,973 (31%) were female. Men treated with immune checkpoint inhibitor monotherapy or combination therapy had a significantly lower risk of mortality than men who received chemotherapy, anti-angiogenesis therapy or other treatments (OS HR: 0.75, 95% CI: 0.71–0.80, *P* < 0.001; Figure 1). In women, the benefit of immune checkpoint inhibitor treatment compared with the control treatment was smaller (OS HR: 0.77, 95% CI: 0.70–0.85; Figure 2). A random model was applied due to substantial heterogeneity among the single-study estimates in men (*I*^2^ = 46.5%, *P* = 0.002) and women (*I*^2^ = 47.0%, *P* = 0.001). The pooled interaction of the OS HR between male and female patients was 0.76 (95% CI: 0.72–0.79, *P* < 0.001).

### PFS analysis

**Figure 3:**
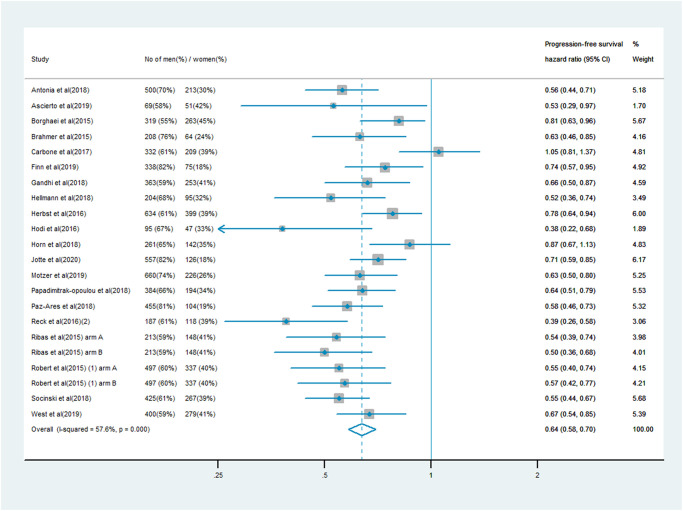
Progression-free survival hazard ratio in male patients in the immune checkpoint inhibitor group compared with the control group.

**Figure 4:**
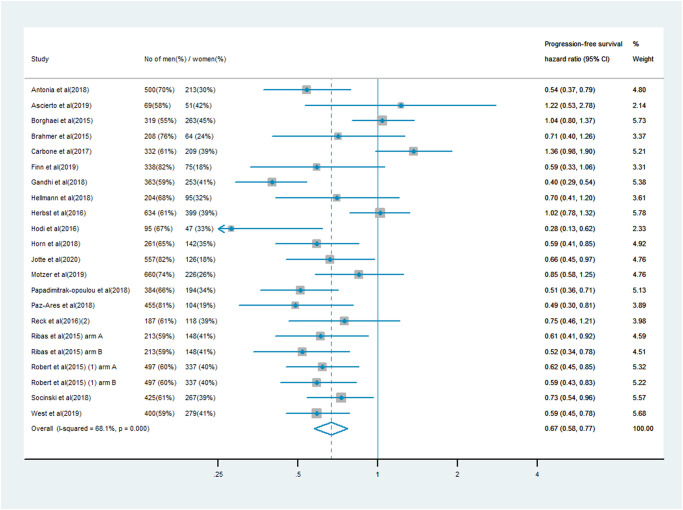
Progression-free survival hazard ratio in female patients in the immune checkpoint inhibitor group compared with the control group.

The analysis of PFS included 10,711 patients, of whom 7,101 (66%) were male and 3,610 (34%) were female. Men treated with immune checkpoint inhibitor monotherapy or combination therapy had a significantly lower risk of mortality than men who received chemotherapy, anti-angiogenesis therapy or other treatments (PFS HR: 0.64, 95% CI: 0.58–0.70; Figure 3). In women, the survival benefit of PD-1/PD-L1 or CTLA-4 inhibitor treatment compared with the control treatment was smaller (PFS HR: 0.67, 95% CI: 0.58–0.77; Figure 4). The pooled interaction of the PFS HR between male and female patients was 0.65 (95% CI: 0.60–0.70, *P* < 0.001). There were no significant differences in the treatment effects of immune checkpoint inhibitors between the sexes, despite the lower pooled HRs for OS and PFS in men (*P* = 0.91 and *P* = 0.80, respectively).

### Subgroup analysis

**Figure 5:**
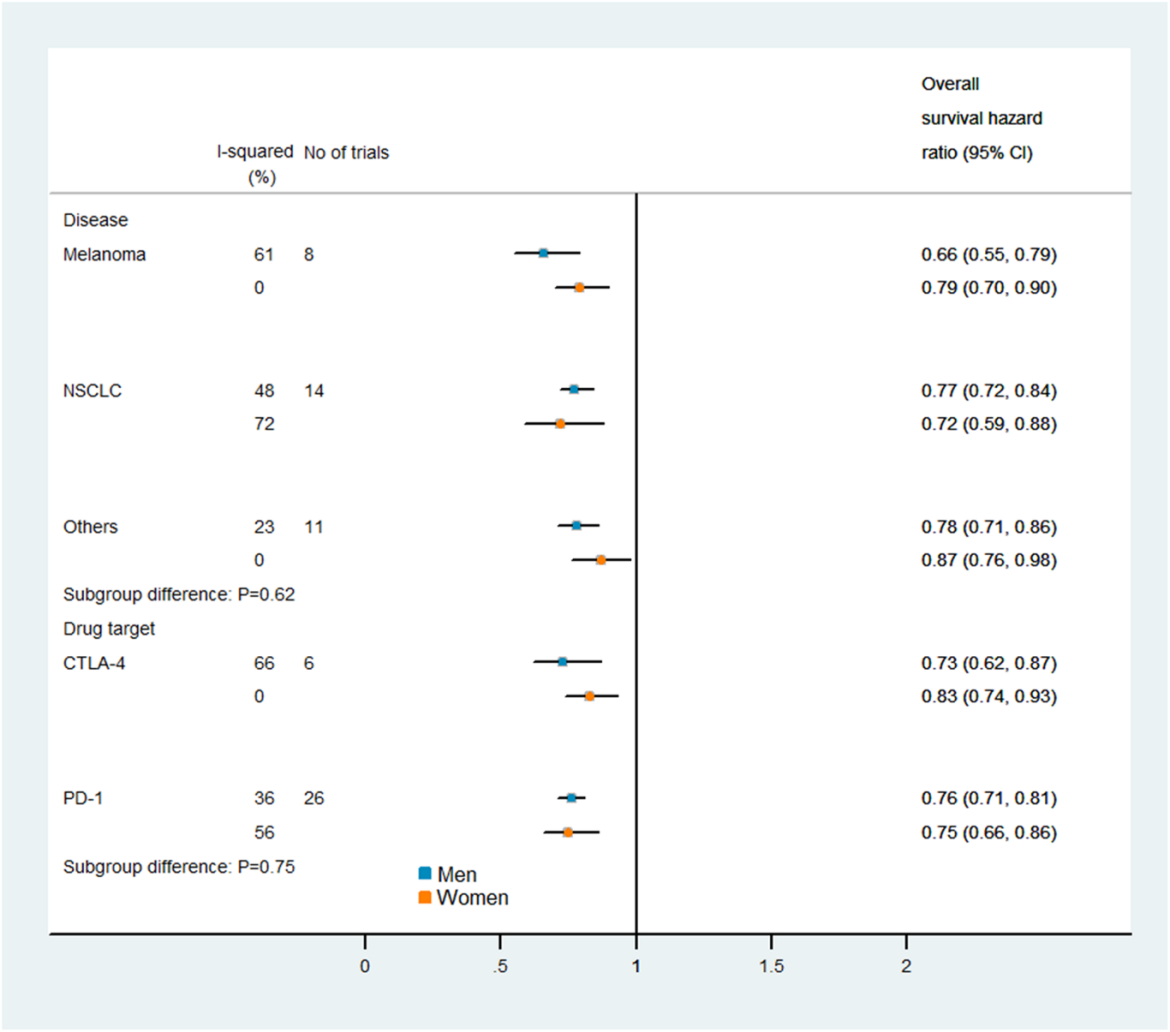
Subgroup analyses of overall survival in patients assigned to the intervention and control groups.

**Figure 6:**
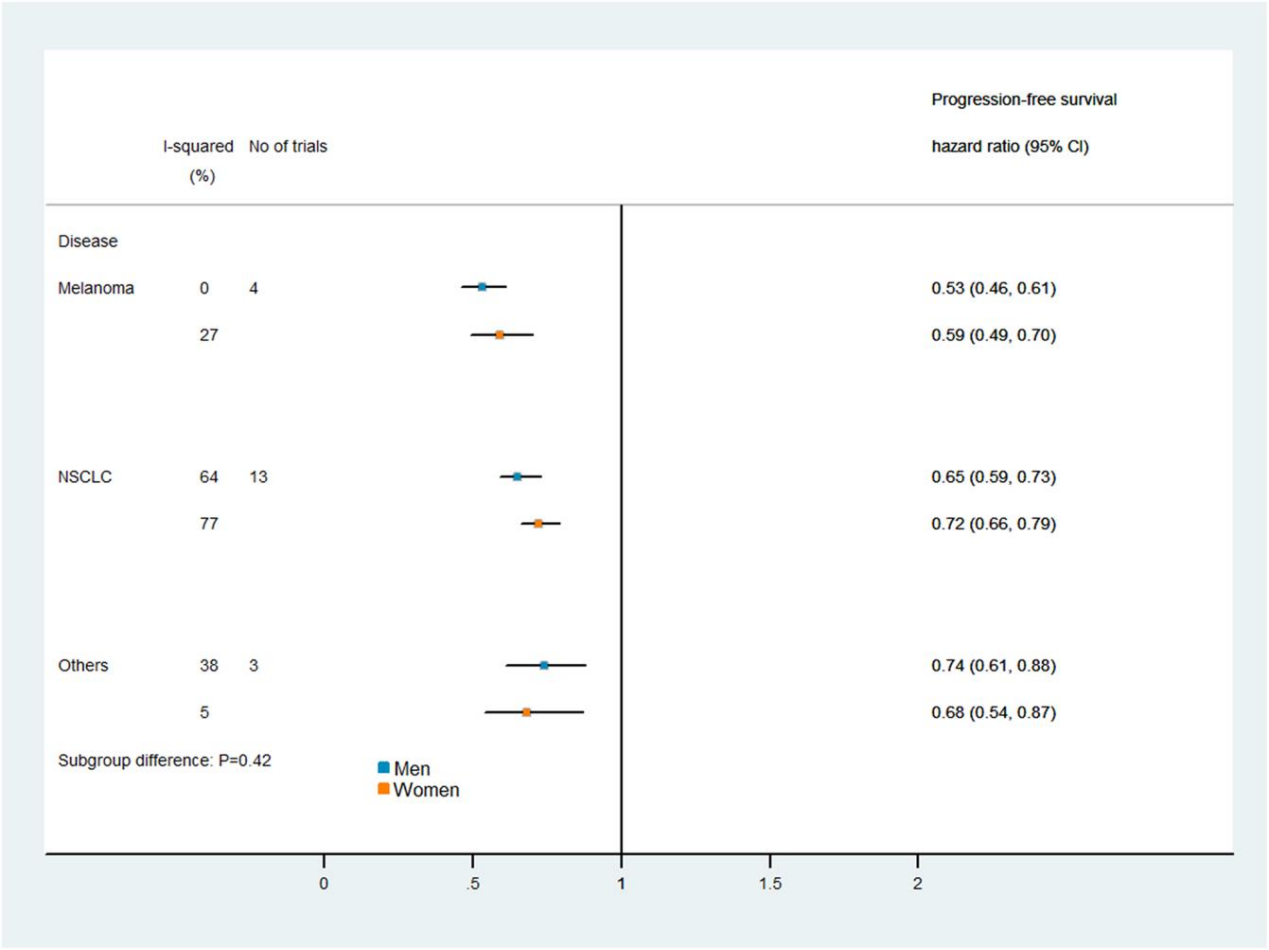
Subgroup analyses of progression-free survival in patients assigned to the intervention and control groups.

Figures 5 and 6 display the results of our subgroup analysis. The subgroup analysis of OS was based on the cancer histological type and the intervention agent target. The subgroup analysis of PFS was only based on the cancer histological type, because 19 of the trials used PD-1 inhibitors, while only one trial used a combination of PD-1 and CTLA-4 inhibitors. Among patients with melanoma, immune checkpoint inhibitors had greater efficacy (i.e., lower pooled HRs) in men than in women. However, the heterogeneity test of the sex-related interactions among the subgroups was not statistically significant.

### Publication bias and sensitivity analyses

To assess potential publication bias, we used the Begg and Egger tests. In the Egger test, the *P*-values for OS and PFS were 0.712 and 0.256, respectively. In the Begg test, the *P*-values for OS and PFS were 0.054 and 0.154, respectively, indicating that there was no publication bias (Supplementary Figures 4–7). To reduce the impact of a single trial on the overall study, we also performed a sensitivity analysis, which suggested that the results of the meta-analysis were robust and reliable (Supplementary Figures 8 and 9).

## DISCUSSION

It has been unclear whether there are sex-related differences in the therapeutic benefits of PD-1/PD-L1 or CTLA-4 inhibitors for cancer treatment. In the current study, we found that the efficacy of immune checkpoint inhibitors compared with control treatments did not differ significantly between men and women. Consistent findings were obtained in subgroup analyses by cancer type and intervention agent target.

Our findings contrasted with those of a previous meta-analysis [17] in which immune checkpoint inhibitors had greater survival benefits for men than for women. Our results also differed from those of a recent study [58] that suggested that influenza vaccines had greater benefits in women than in men, while tumor necrosis factor therapy for rheumatoid arthritis and immune checkpoint inhibitor therapy for tumors had greater therapeutic efficacy in men than in women. These inconsistencies may reflect differences in statistical power and study endpoints. A small sample size, low statistical power and prejudice against the null hypothesis may reduce the probability of determining the true effect and diminish the likelihood that statistically significant results reflect true effects [59, 60].

One advantage of the current analysis was the quality of the available research. Most of the data were from phase III randomized controlled trials. The OS analysis included 19,332 subjects, and the PFS analysis included 10,711 subjects. Our search date was more recent than that of the previous meta-analysis, leading to the addition of 13 large-scale clinical trials. This increased the number of patients by 7,981, including 5,713 (72%) men and 2,268 (28%) women.

In addition, we were able to extend the scope of the study by adding trials on immunotherapy agents such as atezolizumab, durvalumab, avelumab and camrelizumab, which were not included in the previous analysis. Mok et al. [40] compared the survival benefits of pembrolizumab (*n* = 637) and the investigator’s choice of chemotherapy (*n* = 637) as first-line treatments in previously untreated advanced NSCLC patients (KEYNOTE-042). This phase III, open-label clinical trial included 1,274 subjects (902 men and 372 women), was carried out at 213 medical institutions worldwide, lasted over three years (actual study start date: October 30, 2014; actual primary completion date: February 26, 2018), and accordingly contributed significantly to the pooled HR. The PACIFIC trial [38] compared durvalumab (*n* = 476) with placebo treatment (*n* = 237) after chemoradiotherapy in 713 stage III NSCLC patients (500 men and 213 women), and demonstrated that the OS benefit from immune checkpoint inhibitor treatment was greater in female patients (HR: 0.46, 95% CI: 0.30–0.73) than in male patients (HR: 0.78, 95% CI: 0.59–1.03). Huang et al. [46] compared camrelizumab (*n* = 228) with chemotherapy (*n* = 220) as the second-line treatment for advanced/metastatic esophageal cancer in the ESCORT study. The trial involved 400 male and 48 female patients, and revealed a stronger OS benefit in female patients (HR: 0.45, 95% CI: 0.21–0.93) than in male patients (HR: 0.75, 95% CI: 0.60–0.93).

Another strength of our study was the addition of PFS as a study endpoint, which reinforced our conclusions. The PFS analysis included 10,711 participants from 20 trials. The KEYNOTE 189 trial [36] investigated platinum-based chemotherapy plus either pembrolizumab (*n* = 410) or placebo treatment (*n* = 206) in 616 NSCLC patients (363 men and 253 women), and detected a greater PFS benefit in women (HR: 0.40, 95% CI: 0.29–0.54) than in men (HR: 0.66, 95% CI: 0.50–0.87). Similar findings were obtained from the IMpower133 study [39], in which a remarkably greater therapeutic effect was observed in women (HR: 0.59, 95% CI: 0.41–0.85) than in men (HR: 0.87, 95% CI: 0.67–1.13). Clinical trials such as IMpower150, JAVELIN Renal 101 and KEYNOTE-022 [47, 50, 51] used PFS as the research endpoint, but reported no data on OS stratified by sex; thus, the analysis of these studies was essential for detecting the true effect.

Sex-related differences are often reflected in pharmacokinetic differences, but pharmacokinetic differences do not necessarily lead to pharmacodynamic differences. For instance, aspirin tends to be absorbed more quickly by women than by men after oral administration, but its bioavailability does not differ between the sexes [61]. In addition, women have a higher methylprednisolone clearance rate than men, but they are also more sensitive to the drug and have a significantly smaller 50% inhibitory concentration for the inhibition of cortisol secretion, so their net response is similar [62].

Women generally have lower body weights, higher body fat contents and lower muscle contents than men, which may alter organ blood flow and function, thus influencing the pharmacokinetics of many agents [63]. Nevertheless, body weight is directly proportional to the glomerular filtration rate, and men are usually larger than women, so differences in renal excretion rates may well reflect differences in body weight. The renal clearance of fluoroquinolone drugs such as fleroxacin was found to be significantly higher in men than in women, but not after adjustment for weight [64]. Clinical oncology medications (including immune checkpoint inhibitors) are often standardized by body weight or body surface area, which may adjust the pharmacokinetic parameters and offset the differences in pharmacodynamics between men and women.

One limitation of our study was the use of published findings from trials rather than data from individual patients. This prevented us from researching the effects of PD-1 or CTLA-4 inhibitors according to factors such as region, nutritional status or menopausal status. Given their important effects on the immune system, these environmental and hormonal factors are worth investigating [65–68]. In addition, we tested subgroups (male and female patients) in the eligible trials, which may have introduced bias into our results, although the control and intervention arms were balanced in most of the studies. Finally, the differences in OS and PFS between the sexes may have been due to other factors such as comorbidities, age and reproductive status, which were not considered in the trials. Despite these limitations, we believe that our research is valuable, as we systematically assessed data on all the approved immune checkpoint inhibitors and 22,268 patients. To the best of our knowledge, this is the largest meta-analysis of sex differences in immunotherapy responses.

In conclusion, in this up-to-date meta-analysis of all the available studies on immunotherapy in advanced or metastasized cancer, there were no sex differences in the efficacy of PD-1/PD-L1 or CTLA-4 inhibitors. We found no evidence that patient sex should be considered when deciding on the appropriateness of administering immune checkpoint inhibitors to patients with advanced cancers.

## MATERIALS AND METHODS

### Literature search and selection criteria

This study followed the recommendations of the Cochrane Manual for Systematic Reviews of Interventions and the Preferred Reporting Items for Systematic Reviews and Meta-Analyses [69]. The registration number was INPLASY202120071.

We comprehensively searched for phase II and III randomized controlled trials in the PubMed, Embase and Cochrane Library databases from their inception to October 1, 2020. We examined publications from the two major conferences, namely, the European Society of Medical Oncology and the American Society of Clinical Oncology. The keywords were immune checkpoint inhibitors, PD-1, programmed death receptor 1, CTLA-4, cytotoxic T-lymphocyte-associated protein 4, atezolizumab, avelumab, durvalumab, ipilimumab, nivolumab, pembrolizumab and tremelimumab (see Supplementary Materials for details).

The eligibility criteria for inclusion were as follows. First, the clinical trials had to evaluate PD-1/PD-L1 inhibitors, CTLA-4 inhibitors or a combination thereof in advanced carcinoma patients, with the primary outcome measure being the HR for OS or PFS according to the patient’s sex. Second, the trials had to compare immunotherapies with other therapies such as the investigator’s choice of chemotherapy or placebo.

We excluded retrospective studies, prospective observational cohort studies and single-arm phase I and phase II trials (i.e., non-randomized controlled trials). Review articles, case reports, guidelines, conference abstracts, meta-analyses, quality-of-life studies, basic science papers and editorials were also excluded. When a trial was reported in multiple articles or mixed citations, we chose the one with the latest and most complete data. All discrepancies were resolved through discussion. All the randomized controlled trials included in this study represented unique research.

### Risk of bias assessment

The risk of bias was assessed using the Cochrane Collaboration tool [70], which evaluates seven factors: random sequence generation (selection bias), allocation concealment (selection bias), blinding of participants and personnel (performance bias), blinding of outcome assessment (detection bias), incomplete outcome data (attrition bias), selective outcome reporting (reporting bias), and other sources of bias. We examined each study and marked the risk of bias as high, low or unclear. Each low-risk aspect was assigned 1 point, so the highest possible total score was 7 points. Two authors independently evaluated the quality of each article. Differences were resolved through a discussion with all researchers.

### Data analysis

The primary endpoint was the heterogeneity in the efficacy of PD-1/PD-L1 or CTLA-4 inhibitors between male and female patients, determined as the difference in the ln(HR) for OS and/or PFS. Accordingly, for each trial, we extracted the HRs and 95% CIs for OS and PFS separately for men and women. Cochrane’s Q statistic was used to assess the statistical heterogeneity among the different studies and subgroups. *I*^2^ was computed to assess the degree of inconsistency in effect due to heterogeneity among studies. Heterogeneity was defined as high, moderate or low based on *I*^2^ values of 75%, 50% and 25%, respectively. A *P*-value <0.1 indicated greater heterogeneity [71].

The pooled HRs for death in male and female patients were calculated using random effects models. The difference in treatment efficacy between male and female patients was assessed through an interaction test and represented as *P* for the interaction. Subgroup analyses based on the interventional drug target type and cancer type were performed to investigate the influence of sex on the efficacy of immune checkpoint inhibitors in various situations. We only considered subgroups that included more than two trials.

Potential publication bias was assessed using Begg and Egger tests and presented as a funnel plot [72, 73]. All analyses were conducted using Stata version 14.0 (StataCorp, College Station, TX, USA). All *P*-values were two-sided, and *P* < 0.05 was considered statistically significant.

## Supplementary Materials

Supplementary Figures
