# Supplementary material for: Sex-related differences in the efficacy of immune checkpoint inhibitors in malignancy: a systematic review and meta-analysis
Source: Aging (Albany NY). 2021 Jun 4;13(11):15413–32. doi: 10.18632/aging.203100 (PMC8221333; doi:10.18632/aging.203100)
Supplement: Supplementary Figures [file aging-13-203100-s001.pdf]

SUPPLEMENTARY FIGURES

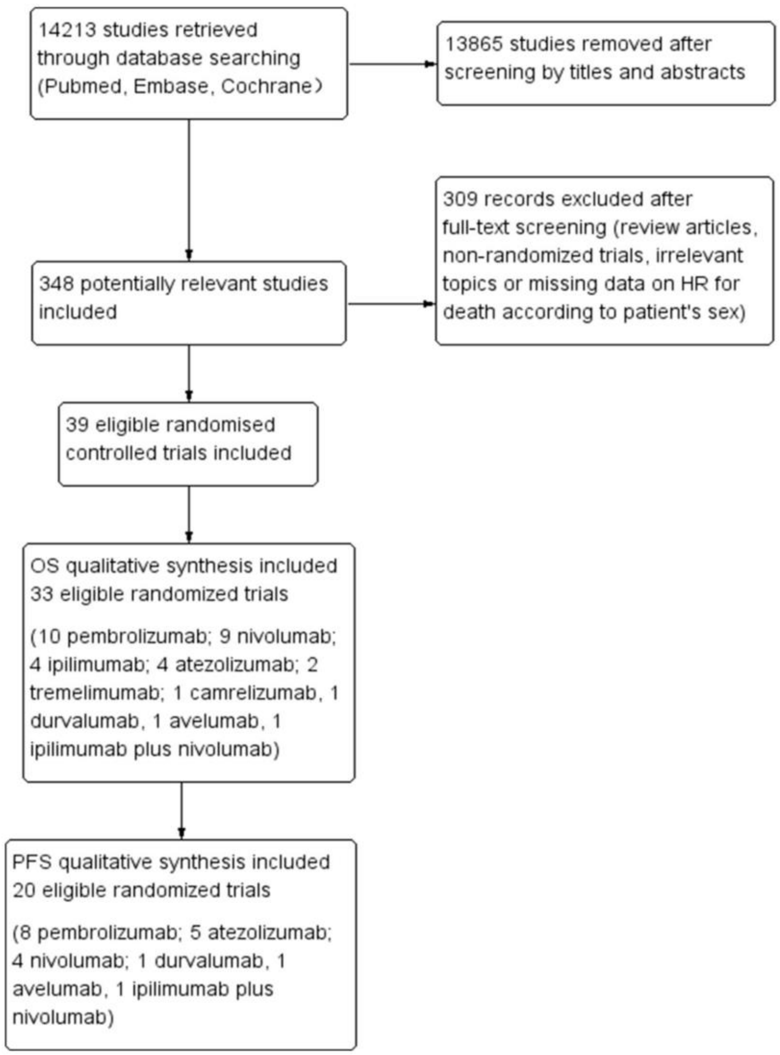

Supplementary Figure 1. The research selection flowchart of this study.

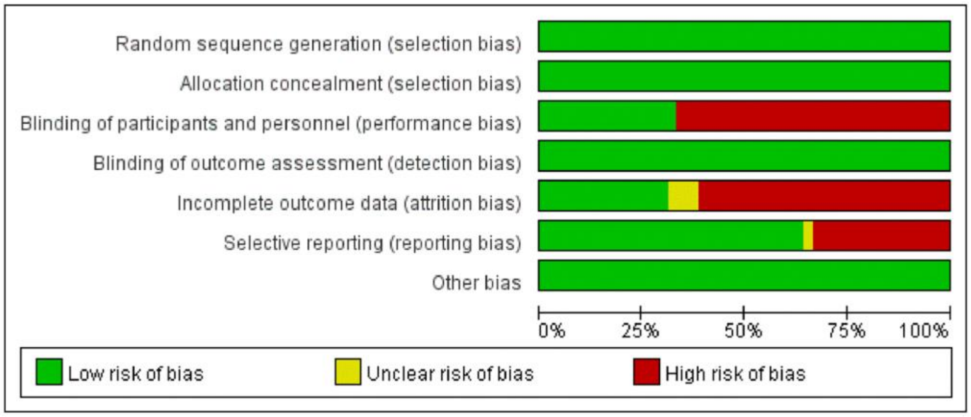

Supplementary Figure 2. Risk of bias graph: judgements about each risk of bias item presented as percentages across all included studies.

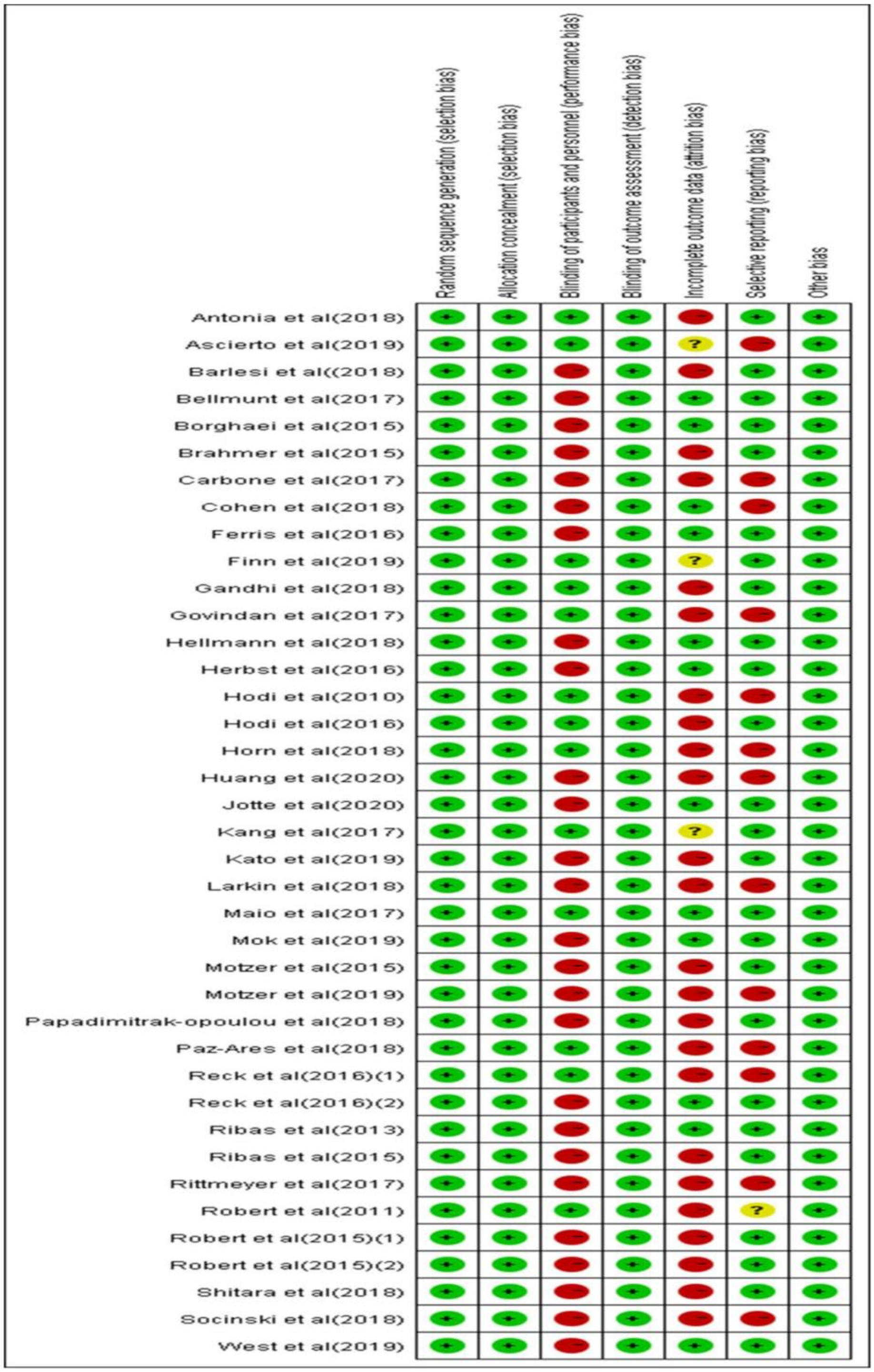

Supplementary Figure 3. Risk of bias summary: judgements about each risk of bias item for each included study.

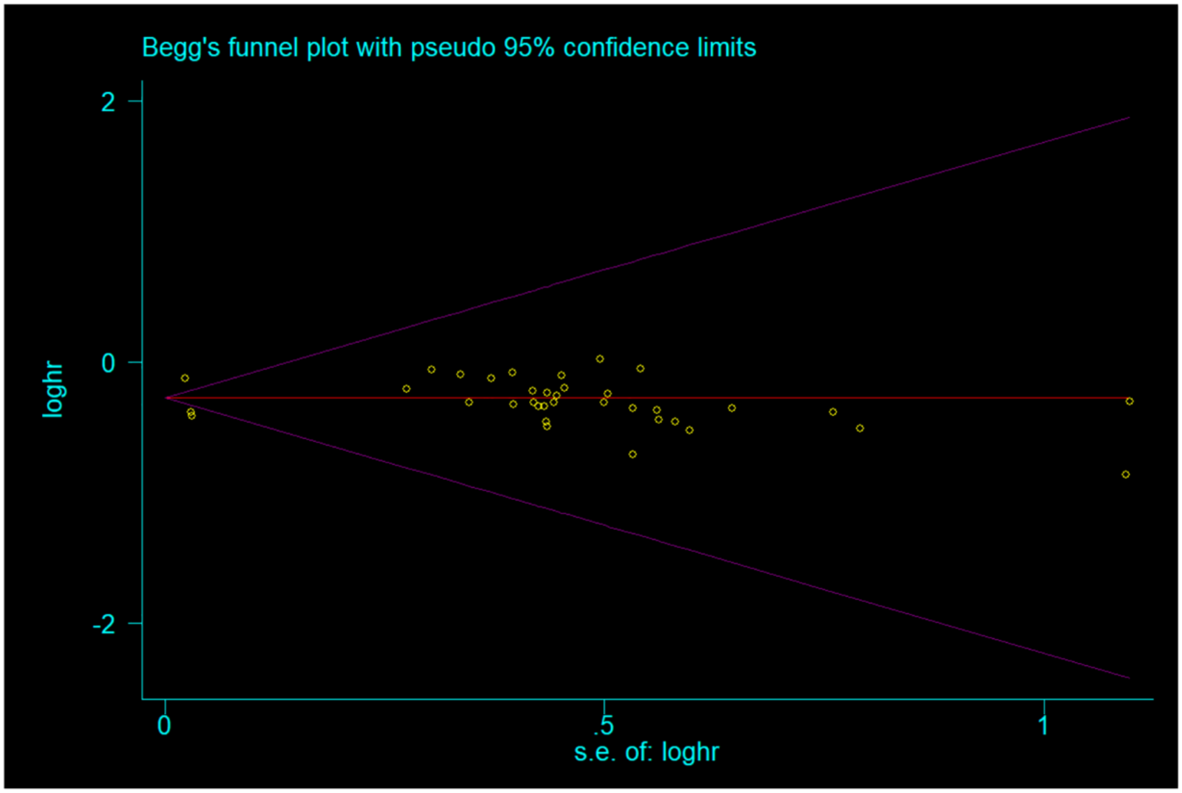

Supplementary Figure 4. The Begg's funnel plot of OS for visual inspection.

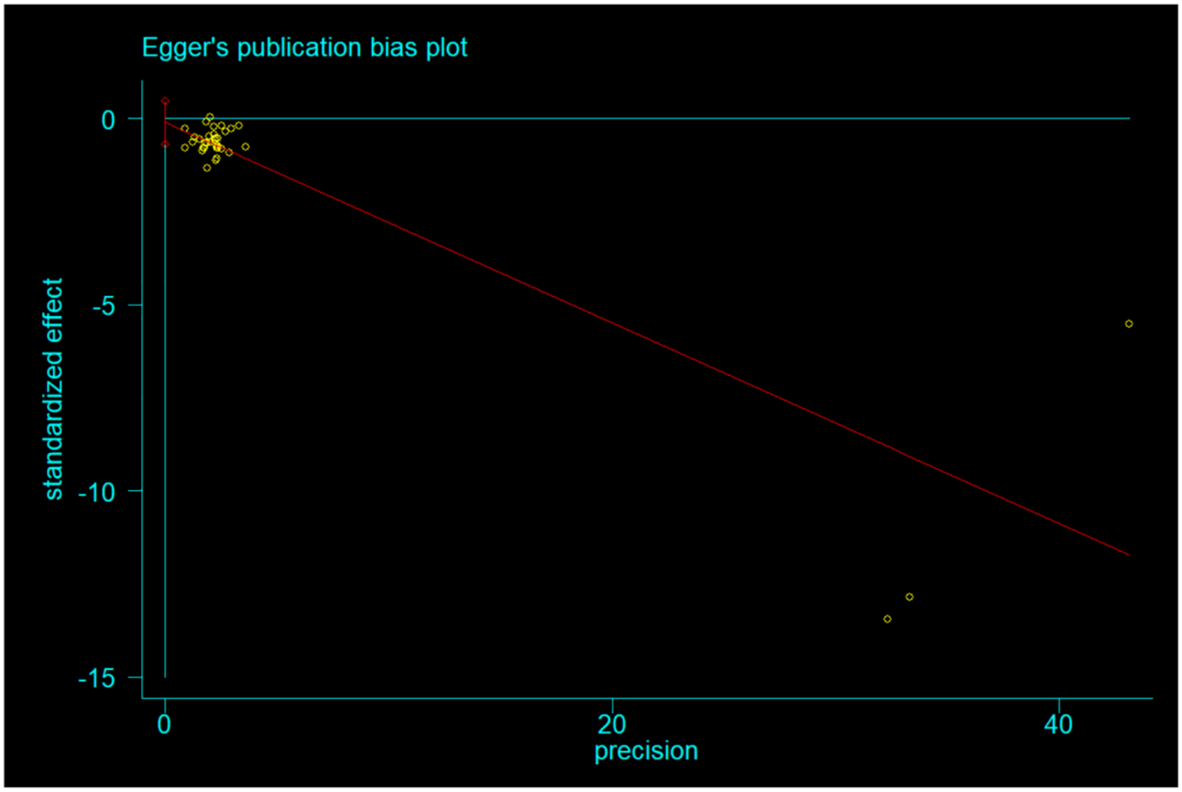

Supplementary Figure 5. The Egger's funnel plot of OS for visual inspection.

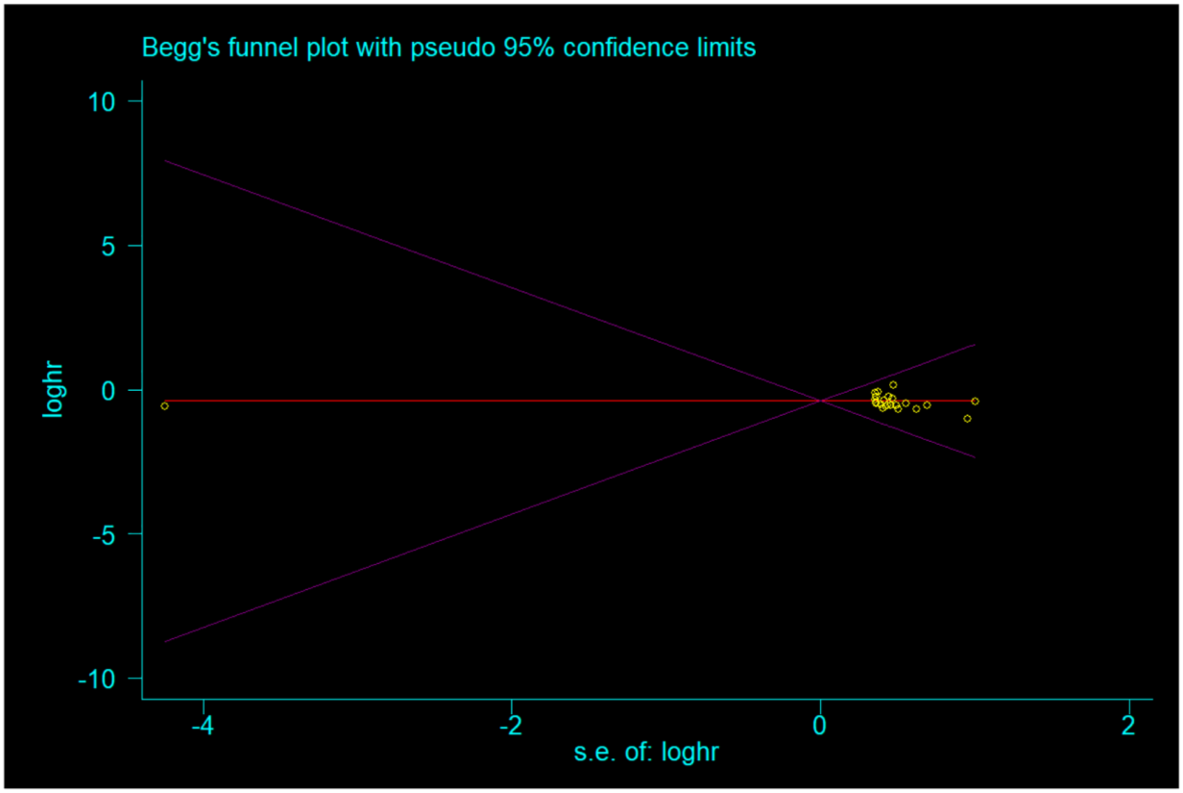

Supplementary Figure 6. The Begg's funnel plot of PFS for visual inspection.

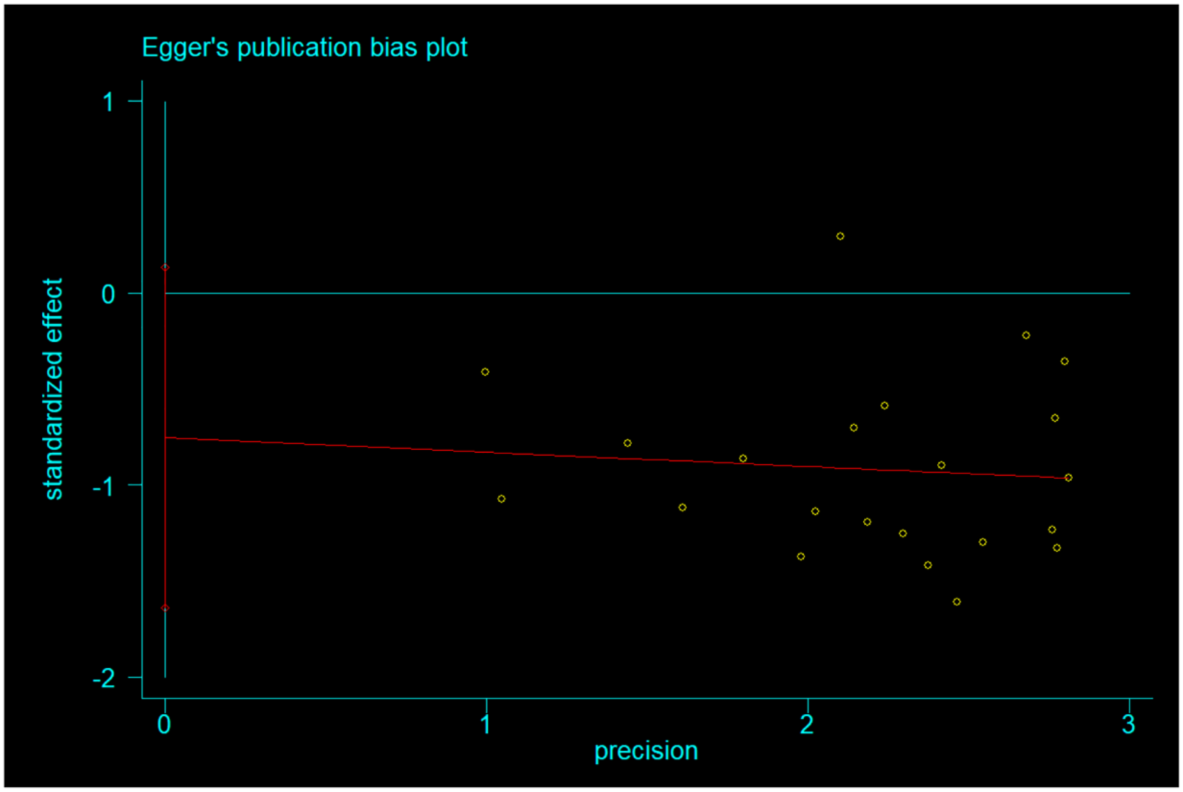

Supplementary Figure 7. The Egger's funnel plot of PFS for visual inspection.

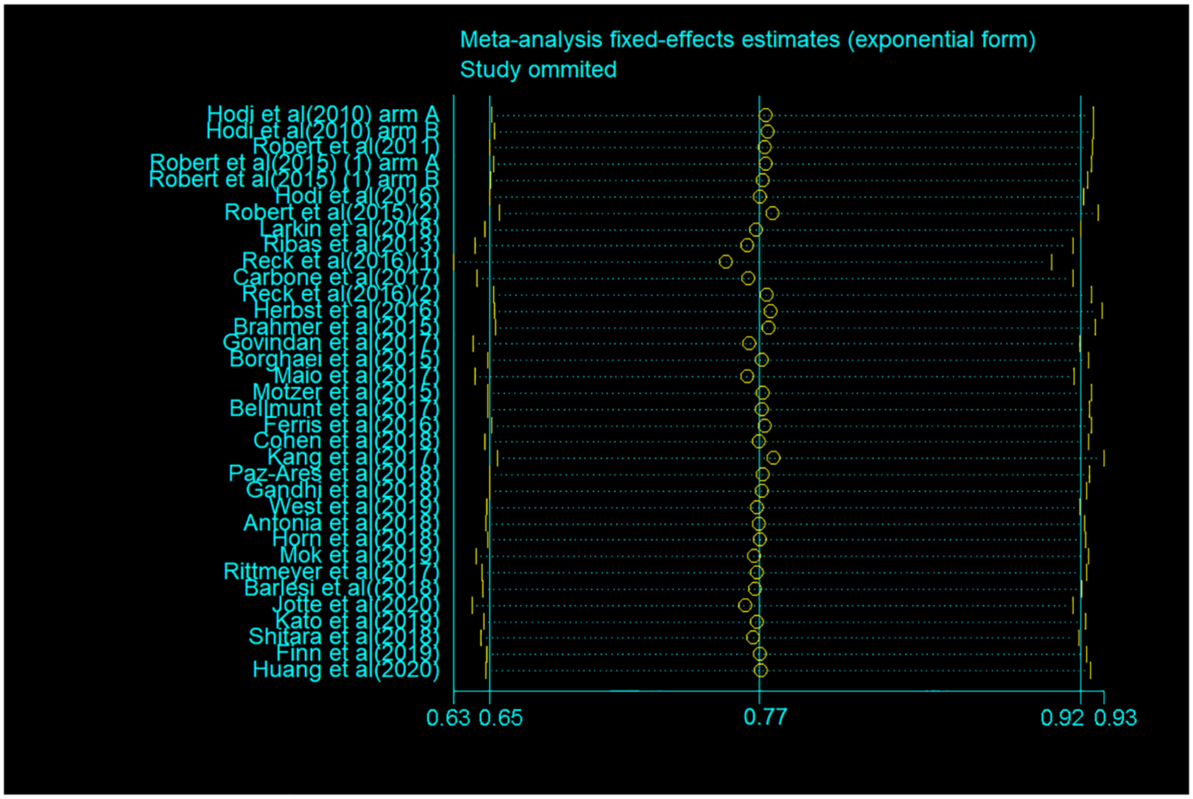

Supplementary Figure 8. Sensitivity analysis using OS as the endpoint of the study.

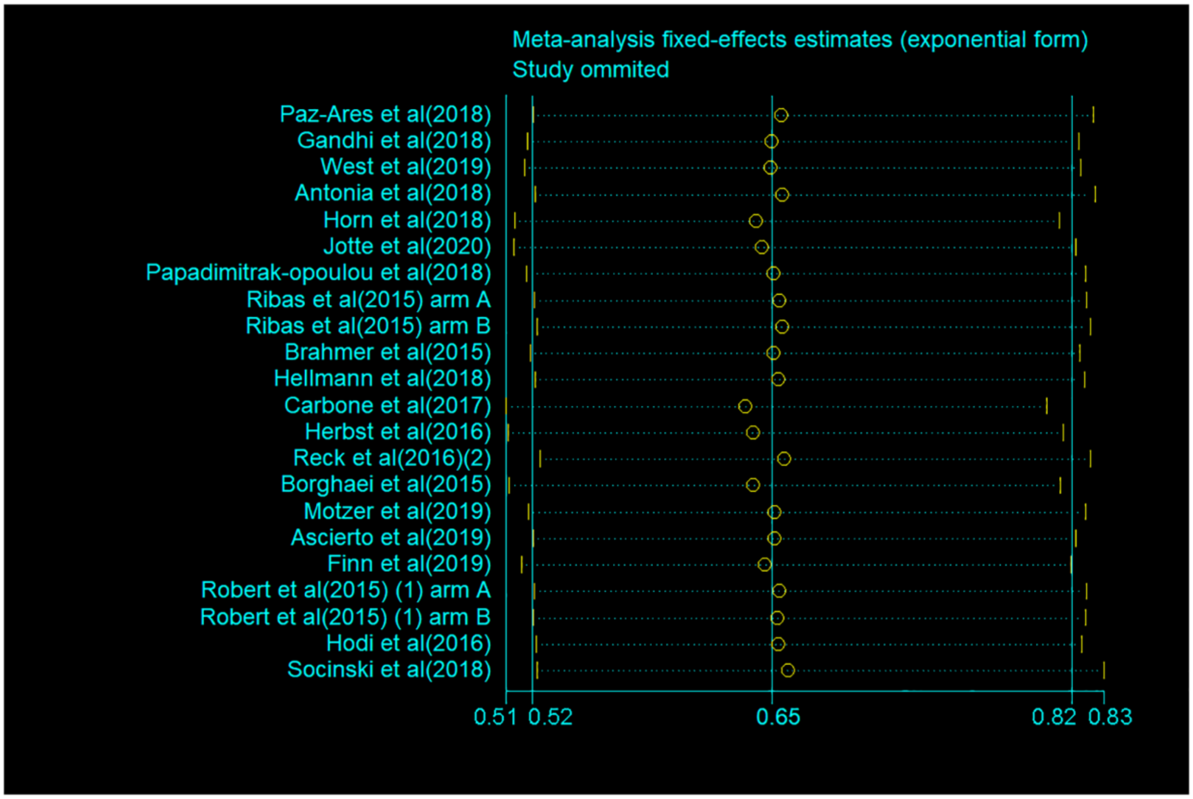

Supplementary Figure 9. Sensitivity analysis using PFS as the endpoint of the study.
